# Colchicine triggered severe rhabdomyolysis after long-term low-dose simvastatin therapy: a case report

**DOI:** 10.1186/s13256-016-1169-z

**Published:** 2017-01-04

**Authors:** Clara Frydrychowicz, Bastian Pasieka, Matthias Pierer, Wolf Mueller, Sirak Petros, Lorenz Weidhase

**Affiliations:** 1 Department of Neuropathology, University Hospital Leipzig, Liebigstrasse 26, House G, 04103 Leipzig, Germany; 2 Medical Intensive Care Unit, University Hospital Leipzig, Leipzig, Germany

**Keywords:** Case report, Rhabdomyolysis, Statin therapy, Anti-HMGCR-antibody, Statin-associated myopathies (SAM), Colchicine

## Abstract

**Background:**

Rhabdomyolysis is a widely recognized yet rare complication in statin use. Rhabdomyolysis might be triggered by the prescription of high doses of statins or by statin accumulation due to interactions with concomitant medication. Muscle cell destruction as evidenced by myoglobin elevation can induce potentially life-threatening acute renal failure.

**Case presentation:**

We report a case of a 70-year-old obese white man with sudden onset of severe rhabdomyolysis with consecutive renal failure. His medication included low-dose simvastatin, which he had taken for 6 years up until the event. The statin was withdrawn immediately. After 3 days of veno-venous hemofiltration his renal function was completely restored.

**Conclusions:**

Clinicians in both primary and special care might be unaware that side effects of statins do occur even after a long uneventful statin medication; they should be advised not to exclude that possibility upfront, even if a patient has tolerated the medication for years.

## Background

Statins are a common group of cholesterol-lowering pharmaceuticals, with the shared pharmacodynamical characteristic of 3-Hydroxy-3-Methylglutaryl-Coenzym-A-Reduktase (3-HMG-CoA) inhibition, the key enzyme in cholesterol synthesis. Inhibition of 3-HMG-CoA by statins is the most effective way of lowering low-density lipoprotein (LDL) cholesterol. Even though adverse effects are frequent, severe complications occur in less than one out of 10,000 patients [1]. Between 2005 and 2011 the US Food and Drug Administration (FDA) Adverse Event Reporting System identified 147,789 case reports with suspected statin-associated myopathy (SAM), including patients with rhabdomyolysis. Rhabdomyolysis as a major statin-associated adverse effect has been reported for all available statins [2] with the highest rate reported for simvastatin [2]. The incidence of clinically relevant rhabdomyolysis is not well defined, but large cohort studies showed incidences of 0.1% [3]. Rhabdomyolysis is defined by creatine kinase (CK) levels at least 10 times normal and reflects acute and massive muscle fiber necrosis accompanied by the release of muscle-related metabolites into the bloodstream [4]. Mortality in general is low with 0.15 deaths per 1 million [5]. Patients with rhabdomyolysis present with muscle weakness, myalgia and brown “tea-colored” urine. Concomitant statin medication in a patient with signs of rhabdomyolysis strongly suggests SAM. Clinical diagnosis without biopsy is possible and depends on multiple symptoms and signs, laboratory findings, and anamnestic characteristics. Rhabdomyolysis with histopathological confirmation has the strongest impact on the diagnosis of SAM. For reference see Table 1. Standard therapy for rhabdomyolysis consists of urine alkalization, aggressive intravenous administration of fluids, and in some cases short-term dialysis and immediate withdrawal of the statin. Here, we report a case of a sudden severe rhabdomyolysis with consecutive renal failure in a patient who received low-dose simvastatin therapy for 6 years without previous complications. This case highlights the difficulty of identifying potential causing or aggravating substances that can lead to SAM and provides high quality histopathological and immunohistochemical pictures of a typical toxic rhabdomyolysis in a patient with statin-induced rhabdomyolysis.

**Table 1:** Diagnosis of statin-induced myopathy

Feature	Points
Symmetrical myalgia	1
Occurrence within 4 weeks from the start of statin therapy	1
Symptoms resolving with withdrawal of therapy	1
Family history of statin-induced myopathy	1
Elevation of creatine kinase	2
Positive re-challenge test	2
Confirmed rhabdomyolysis	5
Histological confirmation of statin-induced myopathy	5

Characteristic features allow classification of patients who have a possible statin-induced myopathy (1–2 points), probable statin-induced myopathy (3–4 points), or definite statin-induced myopathy (>5 points) [9]

## Case presentation

A 70-year-old obese white man presented with acute kidney failure after a 1-year history of progressive muscle weakness and severe generalized myalgia, with difficulty in walking and climbing stairs. His past medical history was significant for chronic renal dysfunction (Kidney Disease Outcomes Quality Initiative IV), chronic heart failure (New York Heart Association III), coronary heart disease with acute myocardial infarction and coronary artery bypass, chronic atrial fibrillation, diabetes type 2, hyperlipoproteinemia, gout, and obstructive sleep apnea syndrome. He had a 6-year history of low-dose simvastatin medication with a daily dose of 40 mg. Further medication included acetylsalicylic acid, rivaroxaban, metoprolol, ramipril, furosemide, molsidomine, isosorbide dinitrate, pantoprazole, and insulin. Colchicine was prescribed as required (0.5 to 1 mg). He had no past medical history of muscular toxicity with statin use.

On admission he was in poor general condition. A clinical examination revealed symmetric proximal muscle weakness of all extremities (level of strength 3/5). Electroneurographic and myographic diagnostics showed chronic myopathic alterations in all of the examined muscles of his upper and lower extremities. Alterations were more pronounced in proximal muscle groups of his lower extremities. He had dyspnea induced by light exercise, edema of lower extremities, no fever, and no rashes.

His CK was elevated with activities >334 μkat/l (Table 2). Antibody diagnostics provided no typical findings for classical antibody-positive autoimmune myositis: dermatomyositis, systemic lupus erythematosus (SLE), scleroderma-myositis overlap syndromes, and Sjögren’s (Sj) antibody-positive autoimmune myositis.

**Table 2:** Patient laboratory data on hospitalization

Parameter	Value	Reference
CK	>334 μkat/l	0.63–2.91 μkat/l
Myoglobin	21896 μg/l	28–72 μg/l
ASAT	4.54 μkat/l	0.17–0.85 μkat/l
LDH	22.54 μkat/l	2.25–3.75 μkat/l
Creatinine	596 μmol/l	59–104 μmol/l
GFR MDRD	8.7 ml/min	
Urea	50.7 mmol/l	<11.9mmol/l
TSH	0.928 mU/l	0.4–3.77 mU/l

*ASAT* aspartate-aminotransferase, *CK* creatine kinase, *GFR* glomerular filtration rate, *LDH* lactate dehydrogenase, *MDRD* Modification of Diet in Renal Disease, *TSH* Thyrotropin

**Fig. 1:**
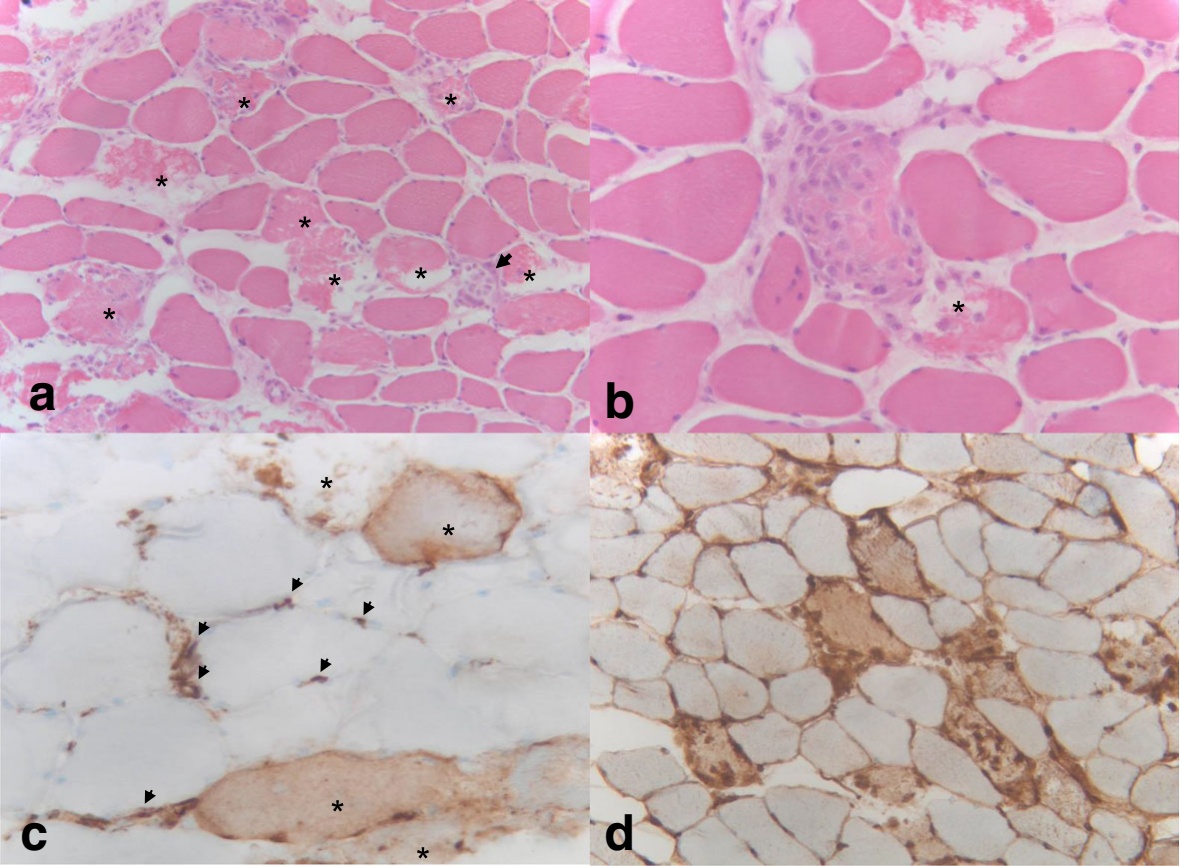
**a** Skeletal muscle with numerous scattered necrotic fibers (*) without signs of inflammation (hematoxylin and eosin, ×100). **b** Single skeletal muscle fiber in a state of myophagocytosis (hematoxylin and eosin, ×200); * marks another fresh single fiber necrosis. **c** Detection of membrane attack complex-positive immune complexes on small vessels (*arrowheads*), endomysial capillaries, sarcolemma, and cytoplasm in necrotic muscle fibers (*) (membrane attack complex, ×200). **d** Clear sarcolemmal and cytoplasmic upregulation of major histocompatibility complex class I (major histocompatibility complex-I, ×100)

Based on the combination of acute kidney failure with muscle weakness and severe generalized myalgia combined with an elevated CK, a muscle biopsy was performed to confirm the diagnosis of SAM and exclude possible differential diagnoses, such as polymyositis. Histology predominantly revealed single fiber necrosis. Necrotic muscle fibers were present throughout the biopsy in a scattered distribution. Some necrotic fibers were in a state of macrophage-mediated degradation (myophagocytosis). Despite widespread scattered muscle fiber necrosis, the tissue was devoid of an interstitial or endomysial inflammatory infiltrate. Immunohistochemistry highlighted a striking sarcolemmal and cytoplasmic upregulation of major histocompatibility complex (MHC) class I expression. Also, complement membrane attack complex (C5b-9)-positive deposits were detected on endothelial cells of endomysial capillaries and on the sarcolemma and in the cytoplasm of necrotic muscle fibers (Fig. 1). The histopathological diagnosis of SAM was made, followed by serum testing for the anti-HMGCR antibody. No anti-HMGCR antibody was detected.

**Fig. 2:**
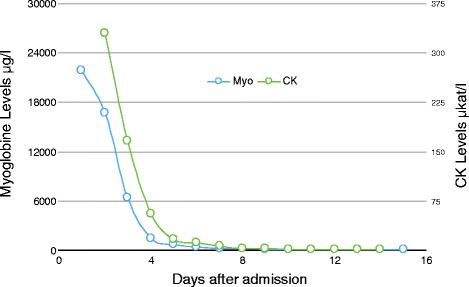
Timely normalization of myoglobin and creatine kinase levels after initiating supportive therapy (continuous veno-venous hemofiltration) and cessation of simvastatin medication. *CK* creatine kinase, *Myo* myoglobin

Acute renal failure, which was induced by muscle necrosis, necessitated a continuous veno-venous hemofiltration (CVVH). Simvastatin was withdrawn and replaced by Ezetrol (ezetimibe). After 3 days of CVVH, his renal function was completely restored. After 15 days his myoglobin and CK levels had normalized (Fig. 2). He was discharged without further complaints on day 18 after hospitalization.

## Discussion

In clinical practice up to 10% of patients with statin medication develop at least mild forms of myopathy [6]. This constitutes an underestimated side effect as was underlined in the Primo Trial; an observational study of muscular symptoms in a randomized population of 7924 patients with hyperlipidemia [6]. In 2012, Germany registered more than 3.2 billion statin prescriptions, which leads to an expectation of a large number of affected or symptomatic patients [7]. For patients with long-term statin usage, a recently published meta-analysis by the Cholesterol Treatment Trialists’ Collaboration states the risk of myopathy to be as low as 0.5 per 1000 patients over 5 years of statin treatment [8]. Almost 30% of statin-associated incidences occur within the first year of treatment [9]. However, onset of muscular side effects has been documented between 2 months and up to 10 years after initiation of statin therapy [4]. Our case confirms and underscores these observations. Six years after well tolerated and uneventful simvastatin medication, our patient suddenly developed a severe statin-associated rhabdomyolysis, which was histologically compatible with the diagnosis of SAM. To date, however, the definition of SAM remains unclear; in particular, the diagnostic criteria of SAM are ill defined, which may explain different data concerning the prevalence and seriousness of SAM [10]. There is general consent that statin can cause several muscle-related complaints. These may range from mild myalgia, may lead to manifest myopathy and myositis-mimicking symptoms, and may culminate in severe rhabdomyolysis. The latter may – when not recognized early enough – induce crush kidney with consecutive renal failure and death [11].

The exact mechanism of SAM still remains elusive. Several theories exist ranging from membrane destabilization due to decreased cholesterol content of skeletal muscle plasma membrane, impaired mitochondrial function due to coenzyme Q10 depletion, disturbed calcium metabolism, and vitamin D deficiency [11]. Also, there are various co-medications that increase the risk of SAM, mostly by interference of the metabolizing cytochrome p450 system (CYP3A4, CYP2C). In particular, the fibric acid derivative gemfibrozil is known to aggravate the symptoms and severity of SAM [12]. The development of statin-associated muscle problems is dose-dependent [10]. However, severe cases of SAM have been reported in patients under low-dose statin medication and without interfering medication. Documented long-term co-medication in the presented case was devoid of potentially interfering drugs. However, personal communication with our patient’s family doctor revealed a newly introduced colchicine medication in close temporal vicinity of clinically manifest rhabdomyolysis. Colchicine was prescribed because muscle pain was misinterpreted as a potential gout attack. Colchicine itself can cause myopathy. Concomitant treatment of colchicine and simvastatin may exacerbate its myotoxic effect (Table 3) [12–15]. Reported case reports of concomitant use of statin and colchicine describe a development of muscle weakness within 8 to 20 days after initiation of colchicine [16–18]. Before excretion, colchicine is metabolized in the liver by demethylation. Statin metabolism may compete with colchicine for the CYP3A4 isoenzyme leading to higher serum concentrations of both medications, thereby increasing the risk of side effects.

**Table 3:** Colchicine triggered simvastatin-induced myopathy: review of the literature

Case reports	Age in years	Sex	Muscle symptoms	Simvastatin	Colchicine
Hsu et al. 2002 [17]	70	M	Symmetrical proximal muscle weakness 3–4/5	*–*	0.5 mg daily
Baker et al. 2004 [18]	79	M	Severe weakness	40 mg daily	0.6 mg daily
Justiniano et al. 2007 [16]	61	F	Rhabdomyolysis	40 mg daily	0.6 mg daily
Sahin et al. 2008 [15]	30	M	Proximal muscle weakness upper and lower extremities 2–3/5	20 mg daily	1.5 mg daily
Oh et al. 2012 [13]	84	M	Symmetrical proximal muscle weakness	40 mg daily	1 mg (first 3 days); 0.5 mg daily
Medani and Wall 2016 [14]	60	M	Muscle weakness 4/5 in all limbs except 5/5 in hip extensor	*–*	1.5 mg daily
Current case	70	M	Rhabdomyolysis	40 mg daily	0.5–1 mg as required

Case reports of concomitant simvastatin and colchicine treatment and development of muscle weakness, including rhabdomyolysis. *F* female, *M* male

Advanced age, female sex, presence of comorbidities, and alcohol consumption are further predisposing factors for SAM [9]. Given the high number of prescriptions, clinicians are in need of reliable risk evaluation methods for the identification of vulnerable patients.

More recently, a self-limiting statin-induced primarily toxic necrotizing myopathy can be distinguished from a persisting autoimmune-mediated statin-induced necrotizing myopathy. The latter persists even after cessation of statin therapy and immunosuppressive therapy [19]. In addition, anti-HMG-CoA reductase antibodies can be detected in the serum and muscle of affected patients [10]. Both SAM and immune-mediated necrotizing myopathy (IMNM) show extensive histopathological overlap and might not be distinguished based on biopsy only. In the present case, IMNM was excluded by antibody testing (anti-HMGCR antibody, negative). Neuropathologists should be aware of the possibility of autoimmune-mediated statin-induced necrotizing myopathy and should recommend anti-HMG-CoA reductase antibody testing in all patients with persistent muscle pathology after statin arrest. Clinical monitoring of SAM may include baseline CK levels of patients especially if there are risk factors such as impaired renal function, genetic myopathy in the past medical history, or significant alcohol abuse [9, 10]. Co-medication should be checked for potential interaction with statins, especially if a new medication is prescribed, including herbal cures. Patients with muscle-related symptoms and statin medication should immediately be checked for an increase in CK. Medication should be stopped immediately and CK levels monitored. In case of persistent elevated CK levels after cessation of statin medication other causes of elevated CK levels should be considered and investigated, including anti-HMGCR-associated SAM [9]. Patients should be informed about the risk of developing myopathy and symptoms. Despite the risk of SAM, statin therapy remains a useful and powerful tool in cardiovascular risk reduction and in reducing cardiovascular morbidity and mortality. Our case emphasizes the need for clinicians to be aware of statin-associated necrotizing myopathy even after long-term statin treatment. The presented patient received uneventful simvastatin therapy for a period of 6 years. In addition, we identified colchicine as a potential trigger of SAM in our patient based on its metabolism potentially competing with statin metabolism. Fast recognition of SAM is mandatory to rescue renal function and avoid life-threatening complications. The treatment of choice remains immediate cessation of statin medication and supportive care for renal function. If recognized early enough, the outcome is excellent.

## Conclusions

We wish to alert clinicians to the fact that side effects of statins do occur even after a long uneventful statin medication; they should be advised not to exclude that possibility upfront, even if a patient has tolerated the medication for years. In particular, for patients with multiple drugs, clinicians should be aware of possible drug–drug interactions such as colchicine and statins. In cases in which colchicine is indicated, statins not metabolized via the CYP3A4 system should be preferred. Patients with muscle-related symptoms and statin medication should immediately be checked for an increase in CK and levels should be monitored after statin medication is stopped. In cases of persistent elevated CK levels after cessation of statin medication, other causes of elevated CK levels should be considered and investigated, including anti-HMGCR-associated SAM. Further studies are needed to clarify the different pathogeneses of statin-induced myopathies, as well as the optimal management of patients with this severe side effect.
